# Personal Health Record Reach in the Veterans Health Administration: A Cross-Sectional Analysis

**DOI:** 10.2196/jmir.3751

**Published:** 2014-12-12

**Authors:** Stephanie Leah Shimada, Cynthia A Brandt, Hua Feng, D Keith McInnes, Sowmya R Rao, James A Rothendler, David A Haggstrom, Erica A Abel, Lisa S Cioffari, Thomas K Houston

**Affiliations:** ^1^Center for Healthcare Organization and Implementation Research / eHealth Quality Enhancement Research InitiativeEdith Nourse Rogers Memorial VA Medical CenterBedford, MAUnited States; ^2^Department of Health Policy and ManagementBoston University School of Public HealthBoston, MAUnited States; ^3^Division of Health Informatics and Implementation ScienceDepartment of Quantitative Health SciencesUniversity of Massachusetts Medical SchoolWorcester, MAUnited States; ^4^Pain Research, Informatics, Multi-morbidities, and Education CenterVA Connecticut Healthcare SystemWest Haven, CTUnited States; ^5^Yale Center for Medical InformaticsYale School of MedicineNew Haven, CTUnited States; ^6^Department of Emergency MedicineYale School of MedicineNew Haven, CTUnited States; ^7^Division of Biostatistics and Health Services ResearchDepartment of Quantitative Health SciencesUniversity of Massachusetts Medical SchoolWorcester, MAUnited States; ^8^Center for Health Information and CommunicationRichard L. Roudebush VA Medical CenterIndianapolis, INUnited States; ^9^Division of General Internal Medicine and GeriatricsDepartment of MedicineIndiana University School of MedicineIndianapolis, INUnited States

**Keywords:** personal health records, patient characteristics, medical conditions, veterans

## Abstract

**Background:**

My Health*e*Vet (MHV) is the personal health record and patient portal developed by the United States Veterans Health Administration (VA). While millions of American veterans have registered for MHV, little is known about how a patient’s health status may affect adoption and use of the personal health record.

**Objective:**

Our aim was to characterize the reach of the VA personal health record by clinical condition.

**Methods:**

This was a cross-sectional analysis of all veterans nationwide with at least one inpatient admission or two outpatient visits between April 2010 and March 2012. We compared adoption (registration, authentication, opt-in to use secure messaging) and use (prescription refill and secure messaging) of MHV in April 2012 across 18 specific clinical conditions prevalent in and of high priority to the VA. We calculated predicted probabilities of adoption by condition using multivariable logistic regression models adjusting for sociodemographics, comorbidities, and clustering of patients within facilities.

**Results:**

Among 6,012,875 veterans, 6.20% were women, 61.45% were Caucasian, and 26.31% resided in rural areas. The mean age was 63.3 years. Nationwide, 18.64% had registered for MHV, 11.06% refilled prescriptions via MHV, and 1.91% used secure messaging with their clinical providers. Results from the multivariable regression suggest that patients with HIV, hyperlipidemia, and spinal cord injury had the highest predicted probabilities of adoption, whereas those with schizophrenia/schizoaffective disorder, alcohol or drug abuse, and stroke had the lowest. Variation was observed across diagnoses in actual (unadjusted) adoption and use, with registration rates ranging from 29.19% of patients with traumatic brain injury to 14.18% of those with schizophrenia/schizoaffective disorder. Some of the variation in actual reach can be explained by facility-level differences in MHV adoption and by differences in patients’ sociodemographic characteristics (eg, age, race, income) by diagnosis.

**Conclusions:**

In this phase of early adoption, opportunities are being missed for those with specific medical conditions that require intensive treatment and self-management, which could be greatly supported by functions of a tethered personal health record.

## Introduction

**Figure 1:**
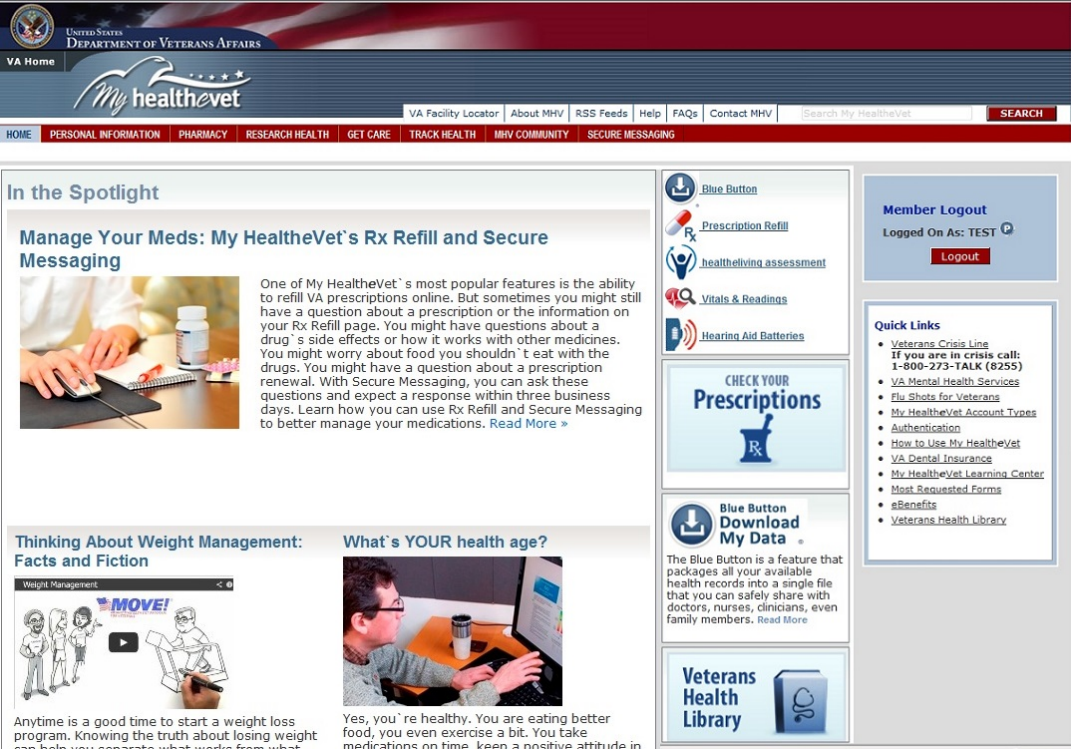
View of MHV home page after login.

The National Committee on Vital and Health Statistics recently recommended adopting the term personal health record (PHR) to refer to “the collection of information about an individual’s health and health care, stored in electronic format” [1]. Although empiric evidence for the effectiveness of PHRs is limited, these systems have the potential to support the transformation of care from episodic visit-based care to continuous, coordinated care [2,3]. PHRs vary significantly in their functionality and content [1,4-6]. My Health*e*Vet (MHV), the Veterans Health Administration’s PHR and patient portal, has multiple levels of access with increasing levels of functionality [7]. Anyone can access health education materials through the website. Those who register for an account can make use of personal health journals, track personal health care information and health measurements (ie, self-enter and track diet, activity, and vital signs), and set personal health care goals [8]. As the PHR is tethered to the US Department of Veterans Affairs (VA) electronic health record (EHR), registrants who are VA patients can also refill prescriptions online. Further, “authenticated” registrants who have also gone through an identity-verification process currently have access to additional EHR-extracted information including their problem lists, medication histories, laboratory results, appointment information, inpatient discharge summaries, and clinical notes. Authenticated patients can also send and receive secure messages with their primary care and specialist teams through MHV. Figure 1 is a screenshot of the MHV home page, showing the options available to users after they log in. New MHV features and functions are constantly being developed and are released periodically with software updates. The above describes the functionality of MHV at the end of 2012. As the PHR of the United States’ largest integrated health care system, MHV has been adopted by over 2.3 million registrants and represents an ideal opportunity to study PHR systems [9].

The potential of PHRs is dependent on the reach of these systems to patients with specific health problems who would most benefit from access to these functions. Reach—the proportion and characteristics of the target population that can access and is willing to use an innovation [10]—is an important concept for evaluation of new technologies [7]. As implementation of PHRs continues to increase, variations in reach of PHRs have been associated with patient characteristics such as age, income, and race [6,11]. Among veterans responding to the 2008 VA Survey of Healthcare Experiences of Patients (SHEP), use of MHV was associated with lower health status [12]. However, the 2010 National Survey of Veterans found that general health status did not significantly predict use of the PHR [13]. Outside the VA, there have also been contradictory reports regarding the association of health status and PHR use, with some studies suggesting that healthier patients with fewer prescriptions were more likely to use PHRs [14,15], while others indicated that sicker patients were more likely to use PHRs [11,16,17]. These contradictory findings suggest a complex relationship between health status and PHR adoption and use that may be dependent on patient characteristics, including demographics (eg, age, gender, race, income, urban vs rural location) and specific medical conditions. Previous articles have proposed that information seeking and engagement in patient technologies may be driven by uncertainty in diagnosis or treatment associated with the patients’ condition, and perhaps condition-specific stigma [18]. Research on PHR adoption and use by patients with varied conditions may help clarify whether patient needs that are specific to each condition (eg, the need for frequent contact with providers, the need for self-management, or the desire to avoid stigma) may drive adoption and use. While there have been a number of studies examining barriers and facilitators of PHR use [19], and the effects of PHR use among groups of patients with specific clinical diagnoses such as diabetes [20] or mental health [21], little research has been conducted to understand differences in PHR reach by patients’ specific clinical diagnoses.

We evaluated the reach of the VA PHR among 6 million US veterans nationwide actively receiving health care from the Veterans Health Administration. The primary goal was to conduct an exploratory analysis to improve our understanding of differences in PHR reach by examining adoption and use of MHV among patients with specific chronic trauma-related, mental, and medical health conditions. We compared the relative reach of MHV using a set of 18 health conditions of high prevalence and/or importance among veterans. We then further explored how patient demographics, comorbidities, and facility characteristics may be affecting MHV reach.

## Methods

### Study Design

We conducted a cross-sectional assessment of PHR adoption and use across clinical conditions. Use was assessed as of April 1, 2012. This study was approved by the Human Research Protection Committees at the VA Connecticut Healthcare System and the Yale School of Medicine, and the Institutional Review Board at the Edith Nourse Rogers VA Hospital in Bedford, Massachusetts.

### Study Population

The study population included all American veterans age 18-100 years who had obtained care from the Veterans Health Administration between April 1, 2010, and March 31, 2012 (N=6,012,875). Cohort inclusion criteria included at least two outpatient visits or one inpatient hospitalization for any cause during this period.

### Data

#### Overview

We used data from the VA system of records available through the VA Corporate Data Warehouse. Variables from the Electronic Health Record included patient demographics and International Classification of Disease (ICD-9-CM) diagnosis codes associated with all VA inpatient and outpatient encounters from October 1, 2007, to March 31, 2012. These data were linked at the patient level with MHV registration, authentication, secure messaging (SM) opt-in, and use log data from April 2012.

#### Definition of Health Conditions (Independent Variables)

We defined patients as having medical and mental health conditions that were coded with ICD-9-CM codes at least once for an inpatient stay or at least twice for outpatient visits. We used previously validated diagnostic code groupings [22] to identify patients with one or more high prevalence or high priority clinical conditions. We selected 18 conditions based on their being high prevalence within the VA population and/or because they were one of the conditions that are high priority for VA clinicians and researchers as evidenced by their inclusion in the VA Quality Enhancement Research Initiative (QUERI) Program, for example, traumatic brain injury (TBI), spinal cord injury (SCI), and human immunodeficiency virus (HIV). Because outpatient codes are commonly assigned by health care providers in the VA and tend to be less accurate than inpatient codes assigned by professional coders, we combined inpatient and outpatient data sources. This has been shown to improve the accuracy of identifying psychiatric disorders [23] and HIV in administrative data [24]. The 18 conditions represented trauma-related (SCI, TBI), mental health (common mood disorders, eg, mild and major depression, anxiety, and posttraumatic stress disorder), substance use disorders (alcohol abuse, drug abuse), other serious mental illnesses (schizophrenia, psychoses), and medical (chronic heart failure, diabetes, hyperlipidemia, hypertension, HIV infection, hepatitis, ischemic heart disease, stroke) clinical conditions.

#### Measuring My Health*e*Vet Reach (Dependent Variables)

Our measures of MHV reach included indicators for adoption of the PHR as of April 2012: (1) registered MHV user (“registered”), (2) registered and in-person authenticated (“authenticated”), and (3) authenticated and opted-in to use secure messaging (“SM opt-in”). Further, we calculated indicators for use of two core PHR functions: (1) use of the online prescription refill feature (“refills”) in MHV, and (2) sending any secure messages to providers (“messages sent”).

#### Sociodemographic Characteristics (Covariates)

Variables were available for the following sociodemographic characteristics: age, gender, race/ethnicity (white, African-American, Latino, other, and unknown), urban/rural residence based on home postal code, and economic need defined as eligibility for free care based on an annual VA financial assessment.

### Statistical Analyses

#### Sociodemographic Characteristics

We first compared the sociodemographic characteristics of veterans across the 18 specific clinical conditions. For sociodemographic characteristics, rates of missing were 0.2% for age, 0.02% for gender, 2.2% for means test, and 4.1% for urban/rural. Only race/ethnicity had a missing data rate of larger than 5%. We categorized the 18.38% of patients with unknown (to patient) or missing race into a group (unknown/missing) and included them in our analysis but excluded patients with missing values on other variables.

#### Main Analysis: Variation of Personal Health Record Use by Clinical Condition

For our main analysis, we assessed reach (adoption and use) of the PHR across the 18 specific clinical conditions. As noted, our main dependent variables were PHR adoption and use. Our main independent variables were the 18 specific clinical conditions. We calculated means, standard deviations, and distributions of veterans’ demographic characteristics, PHR adoption tier (registered, authenticated, opted-in to SM), and indicators of use (messages sent, prescription refills) in all subjects and by clinical condition. To understand the current relative use of the PHR, we then conducted bivariate, unadjusted analyses of the dichotomous MHV adoption and use variables by each condition.

To further understand how complex variations in patient characteristics and facility-level differences in MHV adoption might bias the primary association of specific clinical conditions and MHV adoption, we then obtained adjusted predicted probabilities (adjusted for age, gender, race/ethnicity, economic need via means test, and urban/rural status) and 95% confidence intervals from multivariable models, accounting for clustering of veterans within facilities and including facility as a random effect to adjust for facility-level differences. We ran our multivariable logistic regression models using generalized linear mixed models with a binomial distribution and logit link. We converted the least squares means obtained for each condition from these models into the predicted probabilities (shown as percentages in the tables) using the ILINK option. We also calculated the intraclass correlation coefficient in order to evaluate the potential impact of facility-level variation on the patient-level associations with PHR adoption. A two-sided *P*<.05 was considered to be significant. All analyses were conducted using SAS and SAS Grid 9.2.

To better visualize the result of adjustment for sociodemographic characteristics on the primary association of authentication and clinical condition, we created a multi-attribute plot. This plot shows the association between patient age and economic need, and pre- to post-adjustment change in the relative rank of conditions based on predicted percentages of patients with each condition adopting the PHR. Increasing age and higher economic need were selected for visualization as they are known from prior studies to be aspects of the “digital divide” associated with lower PHR use [11,25].

## Results

### Clinical and Sociodemographic Characteristics

Out of over 6 million (6,012,875) VA patients nationwide, 4,893,286 (81.38%) had one or more of our 18 target conditions, and 1,119,589 (18.62%) had none of the 18 conditions. The most prevalent specific clinical conditions were hypertension (56.63% of patients), hyperlipidemia (55.69%), diabetes (24.71%), and depression (24.68%). The least prevalent conditions were spinal cord injury (0.43%), HIV (0.45%), and TBI (1.40%). Overall, the population had a mean age of 63.3 years and was 6.20% female, 61.45% white, 73.69% urban, and 26.76% were eligible for free care based on a VA financial assessment (see Table 1). Patients without any of the conditions were younger, more likely to be female, urban residents, of unknown race, and less likely to be eligible for free care than patients with at least one condition. See Multimedia Appendix 1 for a table showing demographics by clinical condition.

**Table 1:** Demographic characteristics of patients, overall and by presence of conditions.

Characteristics		Overall (N=6,012,875)	Patients with at least one of 18 conditions^a^ (N=4,893,286)	Patients without any of 18 conditions (N=1,119,589)
Age, mean (SD)		63.32 (16.46)	65.20 (15.02)	55.12 (19.66)
**Gender, %**				
	Female	6.20	5.33	9.98
	Male	93.80	94.67	90.02
**Race, %**				
	White	61.45	62.55	56.66
	African-American	13.09	13.27	12.28
	Latino	5.46	5.39	5.77
	Other	1.62	1.49	2.20
	Unknown/Missing	18.38	17.30	23.09
High economic need^b^, %		26.76	27.30	24.34
**Urban/Rural, %**				
	Urban	73.69	72.73	77.95
	Rural	26.31	27.27	22.05

^a^18 conditions: Medical—hypertension, hyperlipidemia, diabetes, coronary artery disease, chronic obstructive pulmonary disease, congestive heart failure, stroke, hepatitis, HIV; Mental health—anxiety, depression, post-traumatic stress disorder, psychosis, schizophrenia/schizoaffective disorder, alcohol abuse, drug abuse; Trauma-related—traumatic brain injury, spinal cord injury.

^b^Eligible for free care based on a VA financial assessment.

### Variation of Reach (Personal Health Record Adoption and Use) by Clinical Condition

As of April 2012, reach of the PHR remained relatively low. Among patients who had been seen in VA between April 2010 and March 2012, 1.12 million (18.64%) were registered, 0.6 million (10.03%) of these were authenticated, 0.24 million (4.05%) had opted in for secure messaging, and 0.67 million (11.06%) of registered veterans had used the PHR for a prescription refill. Of those opted-in to secure messaging, 47.19% had sent at least one message to their clinical team since opting in. There was significant variation across facilities in PHR adoption. Registration rates varied from 9.30% to 34.91% across facilities (mean 18.82%, SD 5.02), and authentication rates varied from 3.44% to 30.39% (mean 10.76%, SD 4.84).

Reach varied significantly by condition. Table 2 shows the unadjusted breakdown of PHR adoption and use by specific clinical conditions, reflecting actual adoption and use across the VA. Unadjusted adoption was generally higher among patients with trauma-related diagnoses, mood disorders, and posttraumatic stress disorder (PTSD). Veterans with PTSD, TBI, spinal cord injury, depression, anxiety, and HIV more frequently adopted the PHR, compared to those with other conditions. Patients with complex, chronic medical conditions such as hepatitis, coronary artery disease, congestive heart failure, or schizophrenia were less likely to have adopted the PHR.

Higher rates of adoption were also associated with higher levels of use. Among patients with PTSD, 17.01% were authenticated, 18.04% had refilled a medication through the PHR, and 7.00% had opted in to secure messaging, with 3.41% having actually sent a secure message by April 2012. In contrast, 8.05% of patients with schizophrenia/schizoaffective disorder were authenticated, only 8.09% had refilled a prescription through the PHR, and only 1.11% had sent a message. Generally, the reach of the PHR was lower across chronic, complex medical conditions relative to those with trauma-related or mental health diagnoses. Among veterans with coronary artery disease, 11.11% were refilling medications through the PHR, and 1.85% secure messaging, and use was even lower among those with congestive heart failure (10.38% and 1.69%, refills and secure messaging respectively). Those without any of the 18 conditions were considerably less likely than those with one or more diagnoses to have adopted MHV or to have used either of its key features.

**Table 2:** My Health*e*Vet reach (adoption and use) by specific clinical condition^a^.

Condition group (N=6,012,875)	Registered, % (N=1,120,667)	Authenticated, % (N=603,054)	Opted into secure messaging, % (N=243,456)	Sent at least one secure message, % (N=114,884)	Used medication refill, % (N=665,291)
Overall	18.64	10.03	4.05	1.91	11.06
Traumatic brain injury	29.19	16.53	6.39	3.24	17.69
PTSD	28.42	17.01	7.00	3.41	18.04
Spinal cord injury	27.37	16.29	6.65	3.05	18.84
HIV	26.48	15.31	6.30	2.41	17.76
Anxiety	26.39	15.37	6.38	3.16	16.87
Depression	26.17	15.33	6.35	3.15	16.80
Diabetes	21.17	12.52	5.24	2.48	13.60
Hyperlipidemia	20.91	11.76	4.92	2.31	13.20
Psychosis	20.29	11.80	4.38	2.16	12.40
Hypertension	19.70	11.10	4.62	2.17	12.36
Alcohol abuse	19.38	11.18	4.08	1.88	11.38
Drug abuse	18.90	11.21	3.72	1.73	10.32
Chronic obstructive pulmonary disease	18.86	11.04	4.43	2.07	11.86
Hepatitis	18.04	10.89	4.10	1.89	10.16
Coronary artery disease	17.95	10.17	4.11	1.85	11.11
Congestive heart failure	17.22	9.95	3.77	1.69	10.38
Stroke	17.15	9.73	3.68	1.70	10.35
Schizophrenia or schizoaffective disorder	14.18	8.05	2.61	1.11	8.09
None of the diagnoses above	10.81	4.81	1.62	0.73	4.33

^a^These are actual percentages of patients in each condition group, unadjusted for clustering.

### Multivariable Adjustment

Our analyses to explore whether the patient-level findings were influenced by the facility-level adoption of MHV showed significant variation at the facility level (*P*<.001), but this variation did not alter the associations between diagnoses and MHV adoption found at the patient level. Rank ordering of the conditions based on adoption remained largely unchanged after inclusion of facility as a random effect. We found that only 13.7% of the measured variance was at the facility-level, suggesting that the majority of the differences in adoption are predicted by patient characteristics. After inclusion of sociodemographic characteristics and comorbidities, the rank ordering of conditions and the predicted percentage of patients authenticated for MHV use did change (Table 3). HIV, hyperlipidemia, and SCI are the three conditions with the highest predicted percentages of authentication, and both hyperlipidemia and hypertension move up in rank to be among the conditions with the highest predicted percentage of authentication. Younger patients, white patients, women, and patients who did not qualify for free care based on financial need were more likely to be authenticated (results not shown). All sociodemographic variables were significant predictors of authentication; however, age and financial need had the greatest impact on adjusted rates. Authentication rates were adjusted downwards for conditions where patients were younger than average (eg, patients with TBI, PTSD), and upward for conditions where patients tend to be older than average (eg, stroke, coronary artery disease). These effects are mitigated or reversed where a larger-than-average percentage of the population is higher in financial need.

**Table 3:** Unadjusted and adjusted predicted percentages of authentication by specific clinical condition.

Condition group (N=6,012,875)^a^	Unadjusted predicted percentages of authentication (CI)	Adjusted^b^ predicted percentages of authentication (CI)	Difference (adjusted–unadjusted)
HIV	15.33 (14.26-16.46)	16.20 (15.01-17.46)	0.87
Hyperlipidemia	11.78 (10.98-12.64)	16.11 (14.99-17.30)	4.33
Spinal cord injury	16.36 (15.23-17.55)	15.74 (14.58-16.98)	-0.62
Depression	15.21 (14.23-16.26)	15.34 (14.26-16.47)	0.13
Hypertension	11.05 (10.30-11.84)	14.97 (13.92-16.09)	3.92
Post-traumatic stress disorder	16.60 (15.56-17.70)	14.66 (13.62-15.76)	-1.94
Diabetes	12.51 (11.68-13.39)	14.53 (13.50-15.62)	2.02
Anxiety	15.37 (14.37-16.41)	13.95 (12.96-15.01)	-1.42
Traumatic brain injury	16.62 (15.55-17.76)	13.77 (12.77-14.83)	-2.85
Chronic obstructive pulmonary disease	11.08 (10.34-11.88)	13.47 (12.51-14.50)	2.39
Hepatitis	10.64 (9.91-11.41)	13.34 (12.38-14.36)	2.70
Psychosis	11.63 (10.85-12.46)	13.02 (12.09-14.02)	1.39
Coronary artery disease	10.26 (9.56-11.00)	12.78 (11.86-13.76)	2.52
Congestive heart failure	9.89 (9.21-10.61)	12.64 (11.72-13.62)	2.75
Stroke	9.64 (8.97-10.35)	12.37 (11.46-13.33)	2.73
Drug abuse	11.04 (10.29-11.83)	12.29 (11.39-13.24)	1.25
Alcohol abuse	10.90 (10.16-11.68)	12.08 (11.20-13.02)	1.18
Schizophrenia / schizoaffective disorder	8.04 (7.47-8.65)	10.40 (9.62-11.25)	2.36

^a^After excluding patients with missing data for facility or one of the covariates, data for 5,988,043 observations were used for logistic regression analysis. Facility was included as a random effect in both unadjusted and adjusted logistic regression models to adjust for clustering and IPA variation between facilities. For all disease conditions, *P*<.001 in both unadjusted and adjusted logistic regression analyses, except congestive heart failure (*P*=.74) in the unadjusted model.

^b^Adjusted for presence of each other specific clinical condition and demographics including age (continuous), gender, race/ethnicity, economic need, and rural/urban residence. Race/ethnicity includes non-Hispanic white, non-Hispanic African-American, hispanic, other, and unknown/missing with white as reference group.

**Figure 2:**
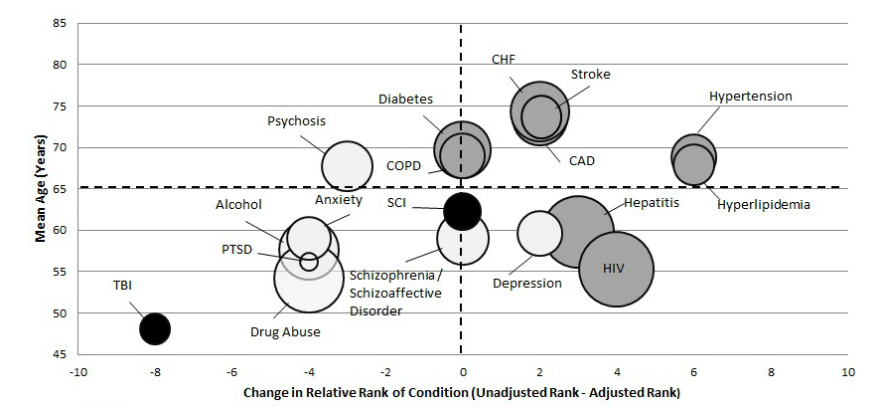
Bubble plot of conditions by mean age (years), change in rank, and socioeconomic status. CAD: coronary heart disease; CHF: congestive heart failure; COPD: chronic obstructive pulmonary disease; HIV: human immunodeficiency virus; PTSD: post-traumatic stress disorder; SCI: spinal cord injury; TBI: traumatic brain injury.

To better visualize effect modification of the association of authentication and clinical condition by age and economic need, we created a multi-attribute plot (see Figure 2). The horizontal axis shows the change in the relative rank after adjustment. The vertical axis is the mean age of veterans in the disease category. Further, the size of the bubbles is proportional to the percent of the population qualifying for free care based on income. The shading of the bubbles distinguishes trauma-related (black), mental health (white), and medical conditions (gray). Using dotted lines, we divided the figure into four quadrants divided by age (above vs below the mean age) and change in rank (above zero=increase in rank after adjustment). For example, we see in the upper right quadrant that all medical conditions affecting older than average patient populations (coronary artery disease, congestive heart failure, chronic obstructive pulmonary disease, stroke, diabetes, hypertension, and hyperlipidemia) have increased in rank post-adjustment.

## Discussion

### Principal Results

As of April 2012, the reach of My Health*e*Vet, a national, multifunction, tethered PHR was less than 20% of veterans actively receiving care in VA, and no clinical group had adoption over 30%. Reach had increased from 16.3% in July 2009, as reported by Nazi et al [7,26] and was slightly lower than the 21.51% estimated by Tsai and Rosenheck [13] based on a survey of 7215 veterans conducted in 2010. During this early phase of adoption, we found that patients with trauma-related conditions and common mental health conditions were among the highest adopters, and patients with certain chronic, complex medical conditions were lower adopters.

Adjusting for sociodemographic characteristics and comorbidities had a significant impact on the predicted probability of authentication. In April 2012, patients with PTSD, TBI, and SCI were the highest adopters of MHV. However, after adjustment, patients with HIV, hyperlipidemia, and SCI were predicted to be the most likely to authenticate. These changes illustrate that differences in actual adoption and use across conditions are at least partially driven by differences in the sociodemographic characteristics of patients by condition. In prior studies, age, ethnicity, and income have been identified as important patient characteristics that predict Internet use in general, and patient portal use more specifically [27]. There were also large facility-level differences in the level of MHV adoption achieved, suggesting that facilities vary meaningfully in the outreach and support they provide to patients regarding adoption and use of MHV. Adjusting for sociodemographic characteristics, comorbidities, and facility-level differences is therefore important when studying PHR reach.

We did find that differences in adoption and use of MHV by health condition remained after adjustment for sociodemographic factors, comorbidities, and facility level variation. All conditions remained significantly associated with authentication in our multivariate model (*P*<.001 for all), and there were statistically significant differences across conditions in the predicted percentages of patients authenticating to MHV. How meaningful those differences are will be determined by the availability of PHR-based tools and interventions to improve self-management and/or health outcomes for patients with that condition. At the same time, the feasibility of developing and implementing such tools and interventions will likely be driven by PHR reach to patients with that condition.

Veterans with trauma-related conditions (TBI and SCI) were frequent PHR users in April 2012. After adjustment, only patients with SCI continued to have a high predicted percentage of authentication. The increased adoption among patients with TBI was largely moderated by their younger age (see Figure 1). Diagnosis of TBI continues to increase [28,29] among young veterans of recent conflicts. The cohort of veterans with TBI is younger, and the relative higher prevalence of adoption, as compared with the other specific clinical conditions, is highly moderated by age. To maximize the effectiveness of the PHR for this group of patients, an adaptive PHR user interface that specifically addresses the cognitive issues in this population may be required. Previous articles have proposed that information seeking and engagement in patient technologies may be driven by uncertainty in diagnosis or treatment associated with the patients’ condition [18]. Because TBI is a condition with varied presentation and uncertain treatment and outcomes, these proposed forces may be driving increased utilization.

Reach of the PHR among patients with depression, anxiety, and PTSD was also relatively high in April 2012. Although a previous study did not detect a significant overall difference in use between veterans receiving care in the VA with and without mental health diagnoses [13], our larger sample size has enabled us to detect differences in actual adoption and use. While these differences are attenuated after adjusting for sociodemographic characteristics, comorbidities, and clustering of patients within facilities, having one of the above mental health diagnoses continues to be a significant predictor of authentication. Patients with depression, anxiety, and PTSD also have higher levels of use of PHRs. A relatively high percentage of these patients had used online prescription refills, and they were among the most likely to have used secure messaging. This was true despite the fact that secure messaging had not yet been implemented among mental health providers and was largely limited to primary care in April 2012. Depression, anxiety, and PTSD are prevalent in the general patient population as well as in VA [28,30-37]. Health care providers have expressed concern that mental health patients would be frequent users of the system, overloading the clinical team with questions and potentially using the system inappropriately. Our results suggest that there may be some reality underlying provider perceptions that patients with specific mental health conditions will frequently use the system. This may be due in part to the fact that patients with multiple medical conditions are more likely to also have mental health conditions [38], thus requiring more health care overall. Future research should address the effectiveness of mental health care via asynchronous secure messaging to assess whether PHRs and other patient-facing technologies improve appropriate access to care or increase inappropriate use.

Among medical conditions, patients with HIV had the highest levels of adoption of the PHR in April 2012, followed by those with diabetes. After adjustment for demographic characteristics, comorbid conditions, and for clustering of patients within facilities, patients with HIV, hyperlipidemia, hypertension, and diabetes had high probabilities of authentication. These are complex conditions, requiring multiple medications and frequent medical follow-up visits. We speculate that higher service utilization and increased need for self-management in these conditions are likely driving PHR adoption. The influence between patient technologies and service utilization may be bi-directional. Patients with more frequent visits to the VA may be more likely to be exposed to information on MHV and encouraged to register and authenticate by their providers and clinic staff. Introduction to PHR functions such as secure messaging can also result in increased service utilization, perhaps by making it easier for patients to raise concerns via online communication with their providers [17]. These conditions also benefit from better nutrition and medication management, as well as improved tracking and monitoring of vitals and readings (eg, blood pressure, blood sugar, weight) and viewing of chemistry/hematology laboratory results (eg, hemoglobin A1c, lipids, CD4+ cell counts, viral load), all of which can be accomplished with assistance from the PHR. In addition, the more medications a patient has, the more potential benefit they may derive from access to the medication refill function of the PHR.

### Limitations

Because the veteran population differs from the general US population in many ways, including higher economic need, higher burden of substance abuse and mental illness, and higher proportion of male patients [29,32,39], these results are not strictly generalizable to non-VA populations. However, it seems likely that adoption and use of PHRs is also driven by clinical need and moderated by patient sociodemographics in non-VA populations [16].

This analysis is limited in the types of PHR use we were able to measure. Understanding the extent of use of other MHV features currently available to veterans, such as medication management tools and wellness reminders, would be of future interest since these features have the potential to improve both medication adherence and evidence-based care [40-42]. In addition, knowledge regarding use of the chronic disease self-management component of MHV is of interest since such features have the potential to significantly improve health outcomes [43-45]. However, at the time of this analysis, our only available measures of “use” were the use of secure messaging or prescription refills. More detailed measures of use are needed to assess the impact of the PHR implementation on outcomes relevant to specific clinical conditions.

After adjusting for patients’ sociodemographic characteristics, their other comorbidities, and the facility at which they received their care, we continued to observe differences in adoption and use of the PHR by diagnosis. However, these analyses were not able to uncover what drives these differences. Also, our analyses also did not focus on patterns of adoption and use among patients with common and/or costly combinations of chronic conditions (eg, diabetes, hyperlipidemia, and hypertension) [46]. Future research should attempt to understand drivers of and barriers to PHR adoption and use.

### Implications for Future Research and PHR Interventions

Variations in adoption and use by diagnosis have implications for delivery of interventions through a PHR or patient portal. Although adoption remained low (less than 30% registered and 17% authenticated in April 2012) for each specific clinical condition, certain groups, such as younger patients with mental illness, may be more ready for and receptive to targeted interventions delivered through a PHR. Understanding the level of adoption and the types of use among patients with the most prevalent clinical conditions can help with prioritizing the development of eHealth tools with the potential to improve self-management and further engage a given patient subgroup.

Our results identified significant gaps in adoption and use. Specifically, reach among patients with certain complex, chronic medical conditions was lower than for those in the high-adoption conditions. Thus, in this phase of early adoption, opportunities are being missed for supporting those with medical conditions that require intensive treatment and self-management via functions of a tethered PHR. These groups should be provided with outreach and supported with interventions including training or educational materials and proactive “help desk” support.

### Conclusions

To our knowledge, this is the first report using national EHR data to associate PHR reach with patient diagnoses. We reported unadjusted reach and then calculated adjusted predicted percentages. Both the unadjusted and adjusted measures have important implications. The unadjusted relative reach reflects the current reality within the Veterans Health Administration and suggests that patients with specific clinical conditions may require additional interventions to support adoption and use. The adjusted estimates provide insights as to the effect of age and economic status on PHR adoption. Although the digital divide of technology access frequently cited in the literature [47] has narrowed, it still exists for older veterans [25] and those of higher economic need [48].

Although an earlier pilot version of My Health*e*Vet had high satisfaction [26] and appeared to improve patient-provider communication and patient engagement in care [49], considerable research needs to be done on the ability of PHRs to support continuous, coordinated, patient-centered, efficient care that is high quality and safe [7]. By first developing interventions for patient populations most ready to adopt them, while providing training and outreach to those groups lagging in adoption, we can move this research agenda forward more rapidly and effectively. Because many patients, both within VA and outside, are multimorbid with mental and medical conditions, future research should also examine how the need to manage multiple comorbid medical and mental health conditions impacts PHR adoption and use.

## Multimedia Appendix 1

Demographic characteristics by condition, ranked by frequency.

## Abbreviations

CAD: coronary artery disease
CHF: congestive heart failure
COPD: chronic obstructive pulmonary disease
EHR: electronic health record
HIV: human immunodeficiency virus
ICD-9-CM: International Classification of Disease, 9th Revision, Clinical Modification
MHV: My Health*e*Vet
PHR: personal health record
PTSD: posttraumatic stress disorder
QUERI: Quality Enhancement Research Initiative
SCI: spinal cord injury
SHEP: Survey of Healthcare Experiences of Patients
TBI: traumatic brain injury
VA: US Department of Veterans Affairs
