# Supplementary material for: Personal Health Record Reach in the Veterans Health Administration: A Cross-Sectional Analysis
Source: J Med Internet Res. 2014 Dec 12;16(12):e272. doi: 10.2196/jmir.3751 (PMC4275468; doi:10.2196/jmir.3751)
Supplement: Supplementary file 1 [file jmir_v16i12e272_app1.pdf]

Multimedia Appendix 1. Demographic characteristics by condition, ranked by frequency.

	Condition N (%)	Age (Mean±SD)	Gender (% Female)	Race (% White)	High Economic Need	Urban/ Rural (% Rural)
Hypertension	3405374 (56.63%)	68.80±12.56	3.56	62.09	27.92	28.43
Hyperlipidemia	3348383 (55.69%)	67.90±12.82	3.86	64.56	25.51	28.97
Diabetes	1485823 (24.71%)	68.95±11.34	2.87	60.92	27.68	28.03
Depression (Major or Minor)	1483734 (24.68%)	59.62±14.76	9.80	61.87	27.20	26.20
Coronary Artery Disease	1252714 (20.83%)	73.48±10.72	1.44	69.16	28.12	30.28
Chronic Obstructive Pulmonary Disease	795707 (13.23%)	69.70±11.56	3.96	69.55	34.95	32.25
Post-Traumatic Stress Disorder	785953 (13.07%)	56.18±15.21	7.91	59.33	11.24	25.94
Anxiety	678242 (11.28%)	58.99±15.73	10.39	65.99	26.74	26.83
Alcohol Abuse	665936 (11.08%)	57.62±12.44	3.65	57.20	37.22	22.89
Psychosis	378498 (6.29%)	67.73±16.41	5.15	61.55	31.22	24.66
Drug Abuse	342284 (5.69%)	54.23±10.90	5.04	47.81	42.53	17.08
Congestive Heart Failure	296207 (4.93%)	74.33±11.25	1.94	66.29	36.57	29.81
Viral Hepatitis	212222 (3.53%)	59.79±7.61	3.11	49.69	44.16	19.01
Stroke	206251 (3.43%)	73.49±11.77	3.03	64.73	33.75	28.13
Schizophrenia or Schizoaffective Disorder	123740 (2.06%)	59.03±11.69	6.92	49.83	32.16	19.19
Traumatic Brain Injury	84299 (1.40%)	48.08±18.12	5.84	63.57	18.64	22.71
Human Immunodeficiency Virus	27033 (0.45%)	55.30±10.35	2.83	35.19	46.00	10.29
Spinal Cord Injury	26046 (0.43%)	62.26±12.77	3.91	62.61	22.66	24.22
None of the Above Diseases	1119589 (18.62%)	55.12±19.66	9.98	56.66	24.34	22.05
